# Burden of disease in adult patients with hereditary angioedema: results from a multinational survey

**DOI:** 10.1186/s13023-025-04134-z

**Published:** 2026-02-19

**Authors:** Maureen Watt, Inmaculada Martinez-Saguer, Angela Simon, Ryan Murphy, Marie De La Cruz, Ricardo Zwiener, Mauricio Sarrazola, Anete S. Grumach

**Affiliations:** 1 Takeda Development Center Americas, Inc., 500 Kendall Street, Lexington, Cambridge, MA 02142 USA; 2 HZRM Hemophilia Center Rhine Main, Frankfurt/Main, Germany; 3 ICON, Raleigh, NC USA; 4 Servicio de Alergia e Inmunología Clínica, Hospital Universitario Austral, Pilar, Buenos Aires Argentina; 5 Departamento de Medicina, Grupo GIPPAM, Universidad de Pamplona, Cúcuta, Colombia; 6 Clinical Immunology, Faculdade de Medicina, Centro Universitario Faculdade de Medicina ABC (CEUFMABC), Santo Andre, Brazil

**Keywords:** Hereditary angioedema, Patient-reported outcomes, Quality of life, Survey

## Abstract

**Background:**

Hereditary angioedema (HAE) is a rare genetic disease manifesting as recurrent painful, burdensome, and potentially life-threatening swelling attacks. This noninterventional, cross-sectional, web-based survey of adult (aged ≥ 18 years) participants with HAE from Argentina, Brazil, Colombia, Croatia, Denmark, Germany, Hungary, Ireland, Norway, Poland, Portugal, Romania, and Sweden sought to deepen the understanding of HAE burden. Individuals were eligible if they had a self-reported physician diagnosis of HAE, ≥ 1 HAE attack or prodromal symptom within the last year, and received HAE medications within the last 2 years. Data were collected on participant demographics, clinical characteristics, and patient-reported outcomes using validated questionnaires; these included disease control (Angioedema Control Test [AECT]), health-related quality of life (HRQoL; Angioedema Quality of Life [AE-QoL]), general health status (12-Item Short Form Survey [SF-12 v2]), anxiety and depression (Hospital Anxiety and Depression Scale [HADS]), and work productivity impairment (Work Productivity and Activity Impairment: General Health [WPAI:GH]).

**Results:**

Overall, 260 participants were included; age (mean ± SD) was 43.3 ± 13.5 years; 72.7% of participants were female, 89.6% had HAE due to C1 inhibitor deficiency, and 78.5% reported family history of HAE. Participants reported 11.5 ± 14.2 (mean ± SD) HAE attacks in the 6 months before the survey, with 68.5% reporting their most recent attack occurring within the last 4 weeks. Of 260 participants, 153 (58.8%) reported currently using any medication for long-term prophylaxis, but only 56/153 (36.6%) reported using a first-line LTP option per international guidelines. Patient-reported disease burden included, on average, moderate to large HRQoL impairment (AE-QoL total score [mean ± SD] 42.9 ± 23.2), poor disease control (AECT score [mean ± SD] 7.4 ± 3.1), and work productivity impairment (WPAI:GH overall work productivity loss score [mean ± SD] 26.9% ± 32.2). Participants with a lower versus higher number of HAE attacks in the past 6 months reported better disease control, less HRQoL impairment, and less work productivity loss.

**Conclusion:**

Results of this large multinational survey highlight that patients included in this study, most of whom were not using first-line LTP, reported being burdened by their disease, including frequent HAE attacks, HRQoL impairment, poor disease control, and work productivity impairment.

**Supplementary Information:**

The online version contains supplementary material available at 10.1186/s13023-025-04134-z.

## Introduction

Hereditary angioedema (HAE) due to C1 inhibitor (C1INH) deficiency (HAE-C1INH, associated with quantitative C1INH deficiency [HAE-C1INH-Type1] or C1INH dysfunction [HAE-C1INH-Type2]) is a rare genetic condition, clinically manifesting as recurrent, unpredictable, and potentially life-threatening attacks of cutaneous or submucosal edema [1, 2]. HAE with normal C1INH (HAE-nC1INH) is rarer than HAE-C1INH and may be caused by mutations in several different genes, only some of which are identified to date [1, 3]. The diagnosed prevalence of HAE is estimated to be 1 in 50,000 to 1 in 100,000 people worldwide [4, 5].

Patients with HAE usually start experiencing symptoms during childhood or adolescence, although some patients experience symptom onset later in life or remain asymptomatic [6, 7]. HAE attacks affect three main areas: skin or subcutaneous tissue (e.g., face, extremities, genitals), abdominal organs, and the upper airway (e.g., larynx or tongue); the frequency and severity of HAE attacks may vary greatly [8]. Laryngeal attacks are of particular concern as they pose a risk of death due to asphyxiation [8]. Although laryngeal attacks have been reported to occur less frequently versus attacks affecting extremities or abdomen, over half of patients with HAE may experience at least one laryngeal attack during their lifetime [7]. The mechanisms underlying the attacks are unclear, and several triggers have been proposed, including minor trauma, medications, infection, or emotional stress [8].

In addition to clinical burden, HAE attacks also negatively impact patients’ health-related quality of life (HRQoL) and pose an economic burden (e.g., reduced work productivity and high healthcare resource utilization [HCRU]) [4, 8–10]. Long-term prophylaxis (LTP) is used to minimize the burden of disease by preventing HAE attacks [1]. Modern, first-line LTP options recommended by the international guidelines on HAE management include C1INH, lanadelumab, and berotralstat [1, 11]; however, the availability of and access to modern HAE treatment options may be limited in some countries [12]. Garadacimab is also approved for LTP in a number of countries, although is not yet recommended by current clinical guidelines. Options for LTP of HAE attacks when first-line medications are unavailable include androgens (e.g., danazol, oxandrolone) and antifibrinolytics (e.g., tranexamic acid) [1, 11, 13].

Several surveys in patients with HAE from Australia, Canada, the United States, and some countries in Europe contributed to a better understanding of HAE and its burden [4, 14–18]. However, key literature gaps remain, especially since previous surveys covered a limited number of geographic locations at a time when a limited number of first-time LTP options were approved and modern LTP options were in their nascent stage. Here, we describe a multinational patient survey that was conducted to describe patient demographics and clinical characteristics and to evaluate the humanistic and economic burden of HAE in adult participants with HAE from expanded geographic locations, many of which were not assessed in previous surveys. The 2021 update to the World Allergy Organization/European Academy of Allergy and Clinical Immunology guidelines for HAE management highlights the importance of patient-reported outcomes (PROs) in assessing HAE activity, impact, and control, and enabling the achievement of HAE treatment goals (complete disease control and the normalization of patients’ lives) [1]. The findings from this burden of disease survey may help to inform decision-making required to understand the range of HAE impacts on patient experience, which in turn would help to inform patient-led treatment choices.

## Methods

### Study design and population

This was a noninterventional, cross-sectional, web-based survey conducted in 13 countries (Argentina, Brazil, Colombia, Croatia, Denmark, Germany, Hungary, Ireland, Norway, Poland, Portugal, Romania, and Sweden) in adult participants with a self-reported physician diagnosis of HAE. The survey on HAE burden in adult patients with HAE was one of three simultaneously conducted surveys in different participant groups (adult patients with HAE, caregivers of adult patients with HAE, and caregivers of pediatric patients with HAE). The questionnaire used in the adult patient survey was adapted from the prior version used in the first wave of the published multinational survey [14]. The protocol was registered at ISRCTN registry (ISRCTN85479564).

Institutional Review Board (IRB) or Independent Ethics Committee (IEC) approval was obtained before recruitment and data collection for each site or country (both central and local ethics committee approval was required in Brazil, Croatia, and Germany). This study was conducted in accordance with the protocol, the Declaration of Helsinki, Good Pharmacoepidemiology Practices, International Society of Pharmacoepidemiology guidelines for Good Pharmacoepidemiology Practices, and local regulations.

Participants in Argentina, Brazil, Colombia, Germany, Ireland, and Portugal were recruited through local healthcare providers, and participants in Croatia, Denmark, Hungary, Norway, Poland, Romania, and Sweden were recruited through the member organizations of the international umbrella organization HAE International (HAEi). Participants were recruited to the survey between July 2022 and February 2023, with a planned recruitment period of 8 weeks in each country. Participants were provided with a brief overview of the study and an individual link to the survey. Participants provided written informed consent before completing the survey; informed consent was collected electronically in all countries except Colombia, where paper informed consent forms with wet ink signatures were requested by the local regulatory authorities.

Key inclusion criteria were self-reported physician diagnosis of HAE (HAE-C1INH-Type1; HAE-C1INH-Type2; HAE-C1INH undifferentiated [participant is unsure of exact HAE type, but it is either HAE-C1INH-Type1 or HAE-C1INH-Type2]; HAE-nC1INH; or unknown [participant does not know what type of HAE they have]); age ≥ 18 years; ≥1 episode of angioedema or prodromal symptoms within the last year; treatment with a prescription medication for an angioedema attack within the last 2 years; ability to understand and provide consent; willingness to complete a web-based survey; and adequate fluency in the local language in which the survey was conducted.

Upon completion of the survey, the participant was provided a unique completion code to email to the recruitment partner (country-specific patient advocacy group, professional organization, medical society, or healthcare provider). Completion codes were used to validate the survey completion and to facilitate sending payment to the individuals for participation (45 USD or local equivalent) in countries where participant remuneration was permitted by local laws and regulations (Denmark, Germany, Hungary, Norway, Romania, and Sweden). Participant remuneration was not permitted by local ethical guidelines and regulations in Argentina, Brazil, Colombia, Croatia, Ireland, Poland, and Portugal.

### Study assessments

The survey included questions on demographics and clinical characteristics, including the number of attacks in the past 6 months, current treatment of HAE, and the participants’ most recent attack (time since their last attack; duration, location, symptoms, and severity of their most recent attack). Attacks were defined as episodes in which the participant experienced symptoms of swelling, abdominal pain, nausea, vomiting, diarrhea, or other symptoms related to their attack.

PROs to describe the participants’ perspective on the humanistic and economic burden of HAE included Angioedema Control Test (AECT), Angioedema Quality of Life (AE-QoL), 12-Item Short Form Health Survey (SF-12 v2), Hospital Anxiety and Depression Scale (HADS), and Work Productivity and Activity Impairment: General Health (WPAI:GH) questionnaires.

AECT is a validated four-question instrument developed to assess angioedema control in patients with recurrent angioedema [19]. The four AECT items include questions on angioedema frequency, angioedema impact on HRQoL, impact of angioedema unpredictability, and perceived angioedema control in the last 3 months [19]. Higher AECT scores reflect patient perception of better disease control; an AECT score of ≥ 10 or < 10 indicates well-controlled or poorly controlled disease, respectively [19, 20].

AE-QoL is a validated, self-administered instrument developed to assess HRQoL impairment in patients with recurrent angioedema over the last 4 weeks [21]. AE-QoL has 17 items divided to four domains (Functioning, Fatigue/Mood, Fears/Shame, and Nutrition); for the scoring, total and domain scores are calculated [21]. Higher AE-QoL domain and total scores reflect a greater HRQoL impairment, with a threshold of ≥ 39 in AE-QoL total score reported to reflect moderate to large effect on HRQoL [21, 22].

SF-12 v2 is a validated instrument developed to measure functional health and well-being over the past week [23]. SF-12 v2 has 12 questions divided to eight health domains (physical functioning, role-physical, bodily pain, general health, vitality, social functioning, role-emotional, and mental health) and two summary scores (Physical Component Summary and Mental Component Summary) [23]. Higher SF-12 v2 scores indicate better general health status, and SF-12 v2 summary scores above and below 50 reflect better and poorer health compared with the general population, respectively [24, 25].

HADS is a self-administered scale developed to assess anxiety, depression, and emotional distress [26]. HADS has 14 items, of which seven relate to anxiety and seven relate to depression in the past week [26]. Higher HADS anxiety and depression subscale scores reflect more severe anxiety and depression, with anxiety and depression subscale scores of 11–14 and 15–21 reflecting moderate and severe anxiety or depression, respectively [26, 27]; a higher HADS score overall reflects greater psychological distress [28].

WPAI:GH is a six-item, self-administered assessment of the effect of health problems on the responder’s ability to work and perform regular activities during the past 7 days [29, 30]. The six WPAI:GH items are used to calculate four scores: percent work time missed due to health (absenteeism), percent impairment while working due to health (presenteeism), percent overall work impairment due to health, and percent activity impairment due to health, with higher scores reflecting a greater productivity loss [29, 30].

Additionally, economic burden associated with HAE as experienced by participants was assessed by evaluating HCRU. PROs and economic burden were evaluated in the overall cohort of participants and in a number of a priori defined subgroups, including subgroups by number of angioedema attacks experienced in the past 6 months (0 attacks, 1–3 attacks, 4–6 attacks, 7–12 attacks, ≥ 13 attacks).

### Statistical analyses

Descriptive analyses were conducted and reported as mean ± standard deviation (SD) for continuous variables and n (%) for categorical variables.

## Results

### Study population

A total of 410 participants were enrolled in the study; of these, 80 did not meet the inclusion criteria, 70 started but did not complete the survey, and 260 completed the survey. Participant demographic and clinical characteristics as well as data on the most recent HAE attack are presented in Table 1. Data for individual countries are reported in Additional file 2: Supplementary Table 1.

**Table 1:** Participant demographic and clinical characteristics

Characteristic	All participants (*N* = 260)
Age, years	
Mean ± SD	43.3 ± 13.5
Range	18.0–81.0
Sex, n (%)	
Female	189 (72.7)
Male	71 (27.3)
HAE type, n (%)	
HAE-C1INH^a^	233 (89.6)
HAE-nC1INH	15 (5.8)
Unknown^b^	12 (4.6)
Age at HAE onset, years, mean ± SD	12.0 ± 8.9
Age at HAE diagnosis, years, mean ± SD	24.2 ± 13.6
Family history of HAE, n (%)	
Yes	204 (78.5)
No	44 (16.9)
Not sure	12 (4.6)
HAE attacks in the past 6 months, mean ± SD	11.5 ± 14.2
Current medication used for LTP,^c^ n/N (%)	
Any	153/260 (58.8)
Androgens	
Danazol	34/153 (22.2)
Oxandrolone	23/153 (15.0)
C1INH	
Human C1INH (Berinert)	24/153 (15.7)
Human C1INH (Cinryze)	1/153 (0.7)
Lanadelumab	24/153 (15.7)
Tranexamic acid	21/153 (13.7)
Berotralstat	10/153 (6.5)
Other	30/153 (19.6)
Missing^d^	107/260 (41.2)
Comorbid conditions,^c^ n (%)	
n with available data	249
Anemia	13/249 (5.2)
Anxiety	54/249 (21.7)
Arthritis	10/249 (4.0)
Asthma	13/249 (5.2)
Cancer	4/249 (1.6)
COPD	1/249 (0.4)
Depression	26/249 (10.4)
Diabetes	15/249 (6.0)
GI disorders	36/249 (14.5)
Heart disease	16/249 (6.4)
High blood pressure	46/249 (18.5)
High cholesterol	27/249 (10.8)
Migraines	29/249 (11.6)
Sleep apnea	6/249 (2.4)
Sleep problems	30/249 (12.0)
Stroke	2/249 (0.8)
Other	75/249 (30.1)
Prefer not to answer	36/249 (14.5)

All data presented in this table are self-reported by survey participants

*C1INH* C1 inhibitor, *COPD* chronic obstructive pulmonary disease, *GI* gastrointestinal, *HAE* hereditary angioedema, *HAE-C1INH* hereditary angioedema due to C1 inhibitor deficiency, *HAE-nC1INH* hereditary angioedema due to normal C1 inhibitor, *LTP* long-term prophylaxis, *SD* standard deviation

^a^Includes participants who answered “HAE Type I”, “HAE Type II”, or “Unsure of exact HAE type, but it is either HAE Type I or II” to the survey question “Which type of HAE do you have?”

^b^Includes participants who answered “I don’t know what type of HAE” to the survey question “Which type of HAE do you have?”

^c^Participants could select more than 1 response (categories nonexclusive) and may be counted in more than 1 category

^d^No response was given to the survey question on current LTP medication

Briefly, most participants were female (72.7%) and had HAE-C1INH (86.3%); HAE-nC1INH was reported by 15 participants, 12 of whom were from Brazil. The age (mean ± SD) at survey completion was 43.3 ± 13.5 years, age at HAE onset was 12.0 ± 8.9 years, and age at HAE diagnosis was 24.2 ± 13.6 years. Of 260 participants, 133 (51.2%) reported HAE onset at < 12 years, 72 (27.7%) participants reported HAE onset at ≥ 12 but < 18 years, and 55 (21.2%) participants reported HAE onset at ≥ 18 years of age. Family history of HAE was reported by 204 of 260 (78.5%) participants, of whom 180 were asked a follow-up question about a family member dying of HAE; 69 of the 180 participants (38.3%) reported deaths in the family due to suffocation as a result of an HAE attack.

In the survey, participants reported 11.5 ± 14.2 HAE attacks (mean ± SD) in the past 6 months. Most participants (178/260; 68.5%) reported that their most recent HAE attack occurred in the last 4 weeks. Data on the severity, duration, and location of the most recent HAE attack were available for 257 participants. The severity of their most recent attack was reported as moderate by 120/257 (46.7%) and as severe by 69/257 (26.8%) participants (Table 2); 106/257 (41.2%) participants reported their most recent HAE attack lasting for longer than 24 h (Table 2). Approximately half of the participants (127/257; 49.4%) reported their most recent attack affecting the abdomen; extremities were also frequently reported, with the participants’ most recent attack affecting the hands in 23.0% (59/257) and feet in 24.5% (63/257) of participants (Additional file 2: Supplementary Table 2). Twenty of 257 (7.8%) participants reported their throat/larynx being affected in the most recent HAE attack, and 45/257 (17.5%) reported their last attack affecting the neck or higher. A wide range of symptoms were reported as occurring during the most recent attack: 127/257 (49.4%) participants reported abdominal pain, 123/257 (47.9%) swelling in extremities, 121/257 (47.1%) abdominal swelling, and 100/257 (38.9%) tiredness (Additional file 2: Supplementary Table 2).

**Table 2:** Timing, duration, severity, location, and symptoms of the most recent HAE attack

Characteristic	All participants (*N* = 260)
Timing of the most recent HAE attack, n (%)	
Within the last 7 days	90 (34.6)
1 to 2 weeks ago	41 (15.8)
2 to 4 weeks ago	47 (18.1)
1 to 2 months ago	33 (12.7)
2 to 6 months ago	34 (13.1)
6 to 12 months ago	12 (4.6)
> 12 months ago/don’t know/can’t remember	3 (1.2)
Duration of the most recent angioedema attack, n/N (%)	
n with available data	257
Up to 6 h	48/257 (18.7)
Between 6 and 12 h	47/257 (18.3)
Between 12 and 24 h or 1 day	54/257 (21.0)
Between 1 and 2 days	45/257 (17.5)
Between 2 and 3 days	41/257 (16.0)
> 3 days	20/257 (7.8)
Don’t know/don’t remember	2/257 (0.8)
Severity of the most recent attack, n/N (%)	
n with available data	257
None	2/257 (0.8)
Very mild	14/257 (5.4)
Mild	42/257 (16.3)
Moderate	120/257 (46.7)
Severe	69/257 (26.8)
Very severe	10/257 (3.9)
Areas of the body affected by the most recent attack,^a^ n/N (%)	
n with available data	257
Trunk	295/257 (114.8)
Extremities	213/257 (82.9)
Face	43/257 (16.7)
Throat and mouth	51/257 (19.8)
Other	3/257 (1.2)

All data presented in this table are self-reported by survey participants

*HAE* hereditary angioedema

^a^Participants could select more than 1 response (categories nonexclusive) and may be counted in more than 1 category. Trunk includes abdomen (stomach), bowels or rectum, buttocks, genitals, chest, side of the body (between chest and hip), bladder, back; Extremities includes feet, hands, legs (including the joints), arms (including the joints); Face includes eyes, cheeks, neck, ears; Throat and mouth includes throat or larynx (voice box), tongue, lips, uvula

### Therapeutic management of HAE

Overall, 171 participants reported having used any on-demand prescription medication to treat their most recent HAE attack and associated symptoms; of these, 85 participants (49.7%) reported using icatibant and 38 (22.2%) reported using human C1-INH as an on-demand treatment to treat their most recent HAE attack. More than half of participants (153/260; 58.8%) reported currently using any medications for LTP; some participants reported currently using ≥ 1 LTP medication. Of 153 participants with current LTP, C1INH, lanadelumab, and/or berotralstat (medications recommended as first-line LTP in the international guidelines) for LTP were reported by only 56 participants (36.6%), three of whom reported currently using two different first-line LTP options each [1, 11]. Androgens (danazol and/or oxandrolone) as the current LTP medication were reported by 56 of 153 participants (36.6%); of these, one participant reported currently using both danazol and oxandrolone. LTP with tranexamic acid was reported by 21 of 153 participants (13.7%). Furthermore, 30 of 153 participants (19.6%) reported using “other” medications (including medications other than C1INH, lanadelumab, berotralstat, androgens, or antifibrinolytics as well as prophylaxis options that were not generally available in a specific country) for LTP. Of 153 participants with current LTP, 82 (53.6%) reported using LTP for ≥ 3 years. Overall, 204 participants responded to the survey question about the satisfaction with the current treatment regimen; the majority of these participants (149 participants; 73.0%) reported being satisfied or very satisfied with their current treatment regimen.

A total of 249 participants answered the survey question about specific health conditions (not including HAE); of these, 54 participants (21.7%) reported having anxiety and 26 (10.4%) reported having depression. Furthermore, 34 participants reported taking prescription medication for anxiety and 19 participants reported taking prescription medication for depression.

### Angioedema control

**Fig. 1:**
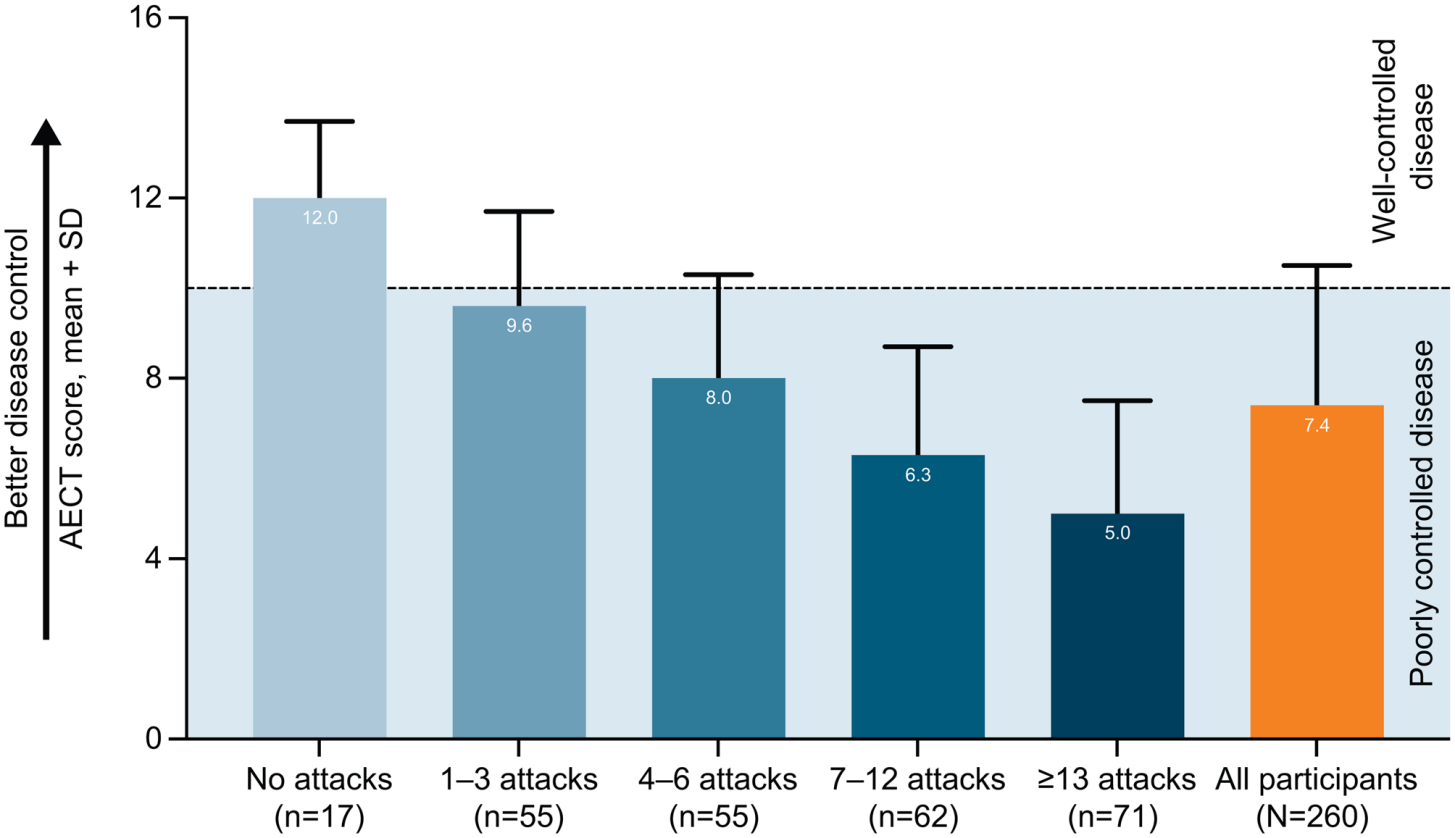
AECT scores by the number of HAE attacks in the previous 6 months. *AECT* Angioedema Control Test, *HAE* hereditary angioedema. Angioedema Control Test (AECT) is a validated, disease-specific measure for patients with recurrent angioedema that assesses disease control which consists of 4 questions over a recall period “in the last 3 months” [19]. The AECT score has a scale of 0–16, where higher scores indicate patient perception of better angioedema control, and is calculated by summing the 4 item scores [19]. AECT score thresholds of < 10 and ≥ 10 indicate poorly and well-controlled recurrent angioedema [20]

Angioedema control assessed with the AECT in the overall cohort is shown in Fig. 1; data by country are reported in Additional file 2: Supplementary Table 3. The AECT score (mean ± SD) was 7.4 ± 3.1 in the overall cohort, in which 195 of 260 participants (75.0%) had an AECT score < 10, highlighting that the majority of the respondents did not perceive their disease to be controlled. In participants who reported currently taking LTP, the AECT score (mean ± SD) was 8.1 ± 2.9, while in participants who did not report taking any LTP, it was 6.4 ± 3.0. Of 260 participants, 111 (42.7%) reported that unpredictability of their angioedema bothered them much or very much in the past 3 months in the response to the AECT question on angioedema bothersomeness (a little bothered: 64/260, 24.6%; somewhat bothered: 50/260, 19.2%; much bothered, 67/260, 25.8%; very much bothered: 44/260, 16.9%). Additionally, only 21.9% of participants (57/260) reported that their angioedema was often or very often controlled by their therapy in the past 3 months in the response to the AECT question on angioedema control.

Participants who experienced a lower versus higher number of HAE attacks in the past 6 months reported numerically higher AECT scores (Fig. 1), indicating that participants with lower versus higher attack frequency reported a better perception of disease control. The mean AECT score was ≥ 10 (over the threshold score indicating well-controlled disease) only in participants with no HAE attacks in the past 6 months. No clear relationship was observed between the number of HAE attacks in the past 6 months and the participant responses to the AECT question on how well their angioedema was controlled by their therapy. Indeed, the proportion of participants reporting that their angioedema was often or very often controlled by their therapy was lowest in subgroup of participants with 1–3 attacks in the past 6 months (8/55 patients; 14.5%) and highest in participants with 7–12 attacks (17/62 patients; 27.4%).

**Fig. 2:**
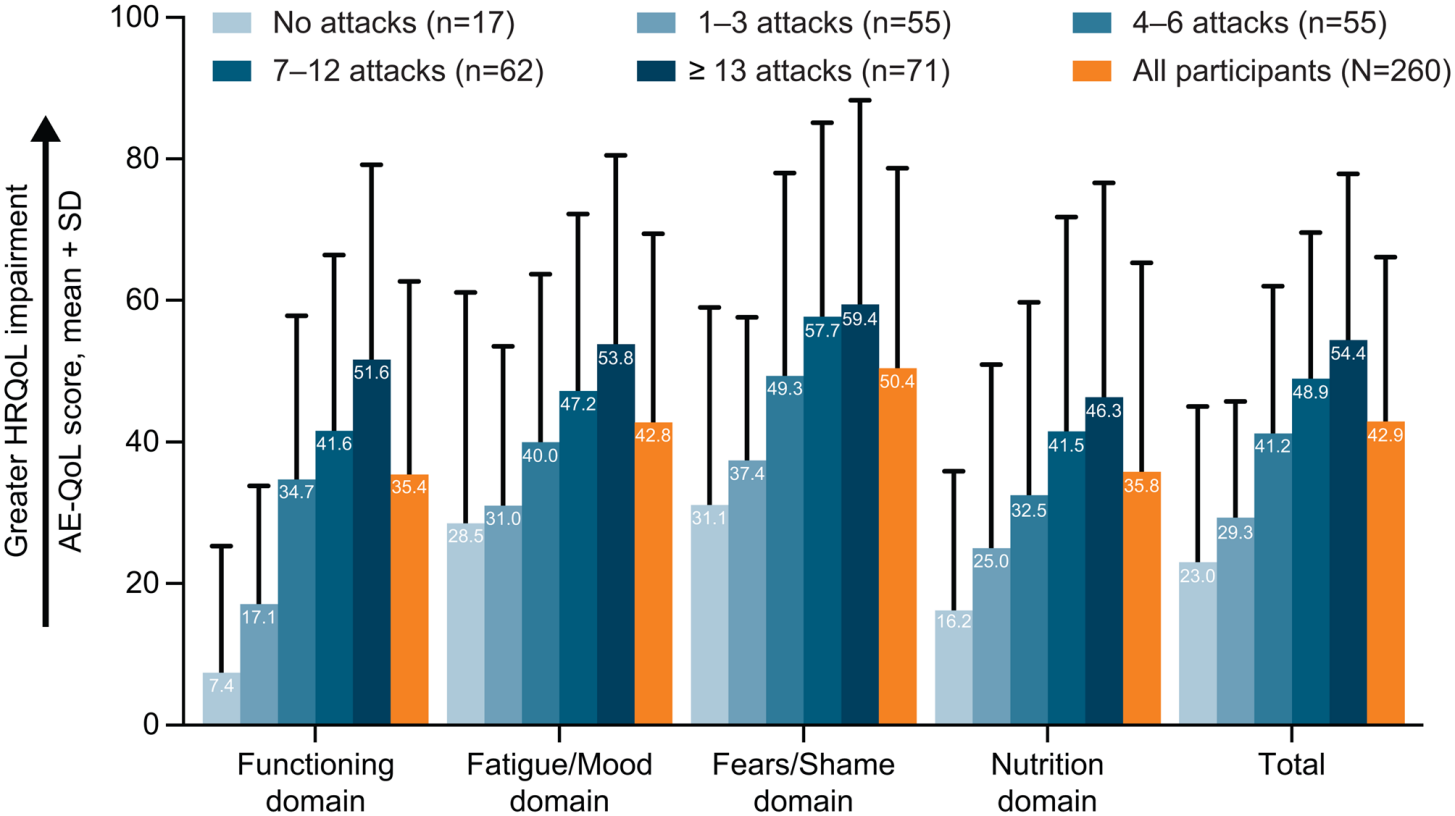
AE-QoL total and domain scores by the number of HAE attacks in the previous 6 months. *AE-QoL* Angioedema Quality of-Life, *HAE* hereditary angioedema, *HRQoL* health-related quality of life. Angioedema Quality of Life (AE-QoL) questionnaire is a validated, disease-specific measure for patients with recurrent angioedema that assesses health-related quality of life (HRQoL) impairment which consists of 17 questions in 4 domains (Functioning, 4 questions; Fatigue/Mood, 5 questions; Fears/Shame, 6 questions; Nutrition, 2 questions) and a total score over a recall period “over the past 4 weeks” [21]. The AE-QoL domain scores and total score have a scale of 0–100, where higher scores indicate a greater HRQoL impairment [21]. AE-QoL total score thresholds of 0–23, 24–38, and ≥ 39 were proposed to define no effect, small effect, and moderate to large effect of recurrent angioedema on the patients’ HRQoL [22]. A 6-point difference in the AE-QoL total score has been validated as a minimal clinically important difference [44]

**Fig. 3:**
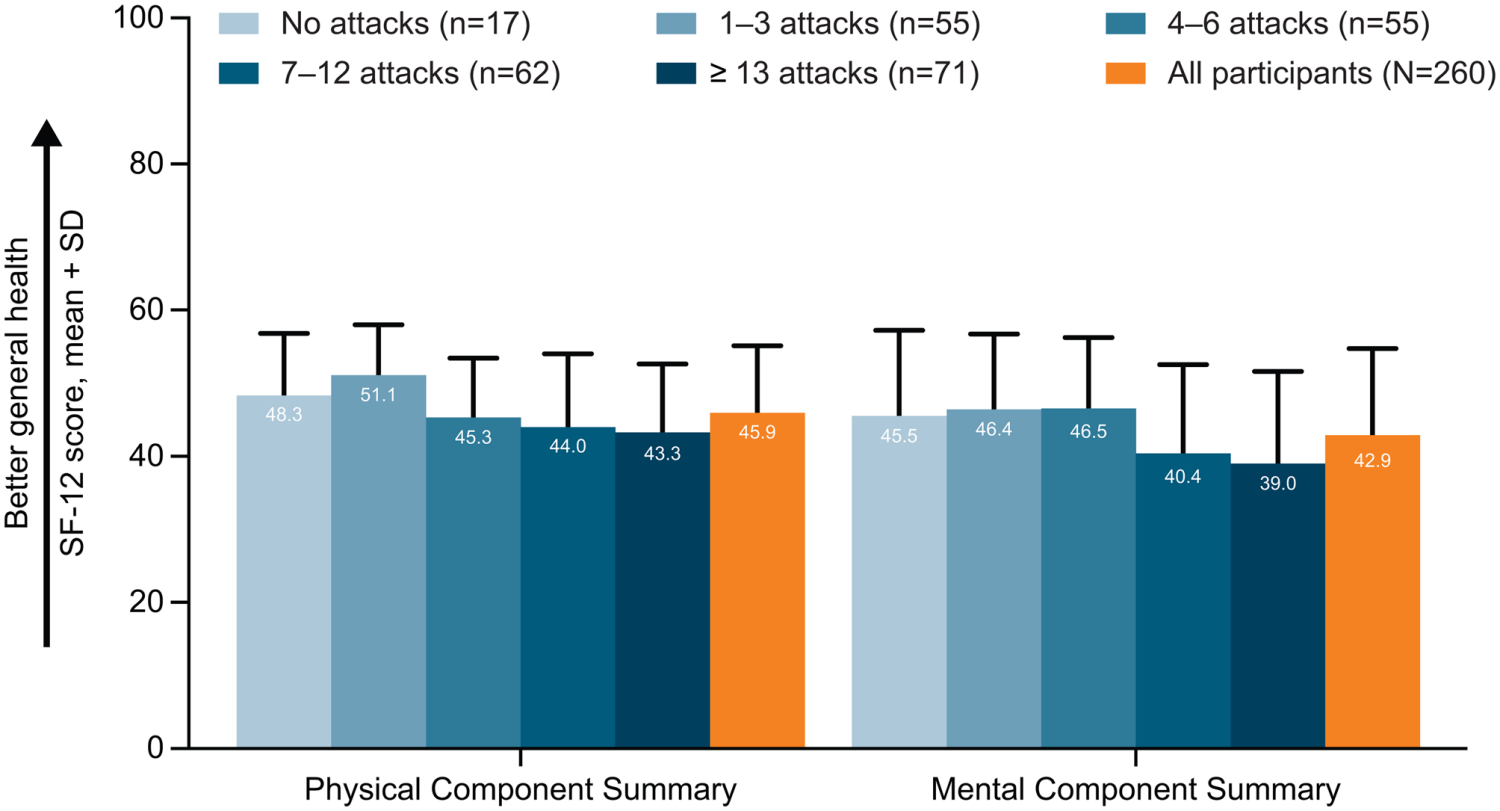
SF-12 Physical Component Summary and Mental Component Summary scores by the number of HAE attacks in the previous 6 months. *HAE* hereditary angioedema, *SF-12* 12-Item Short Form Health Survey. 12-Item Short Form Health Survey (SF-12 v2) is a generic measure that assesses general health, which consists of 12 questions in 8 domains (physical functioning, 2 questions; role-physical, 2 questions; bodily pain, 1 question; general health, 1 question; vitality, 1 question; social functioning, 1 question; role-emotional, 2 questions; mental health, 2 questions) over the recall period “over the past week” [23]. Subscale scores have a scale of 0–100, with higher scores indicating lower impairment [25]. Additionally, SF-12 v2 has two summary scores: Physical Component Summary and Mental Component Summary, which are calculated from the responses to 12 questions by applying physical and mental regression weights (derived from the general US population), respectively [24]. In the general US population, both SF-12 v2 Physical and Mental Component Summary scores have a mean of 50 with a standard deviation of 10; scores above and below 50 indicate physical or mental health that is above or below that of the general US population [24]

**Fig. 4:**
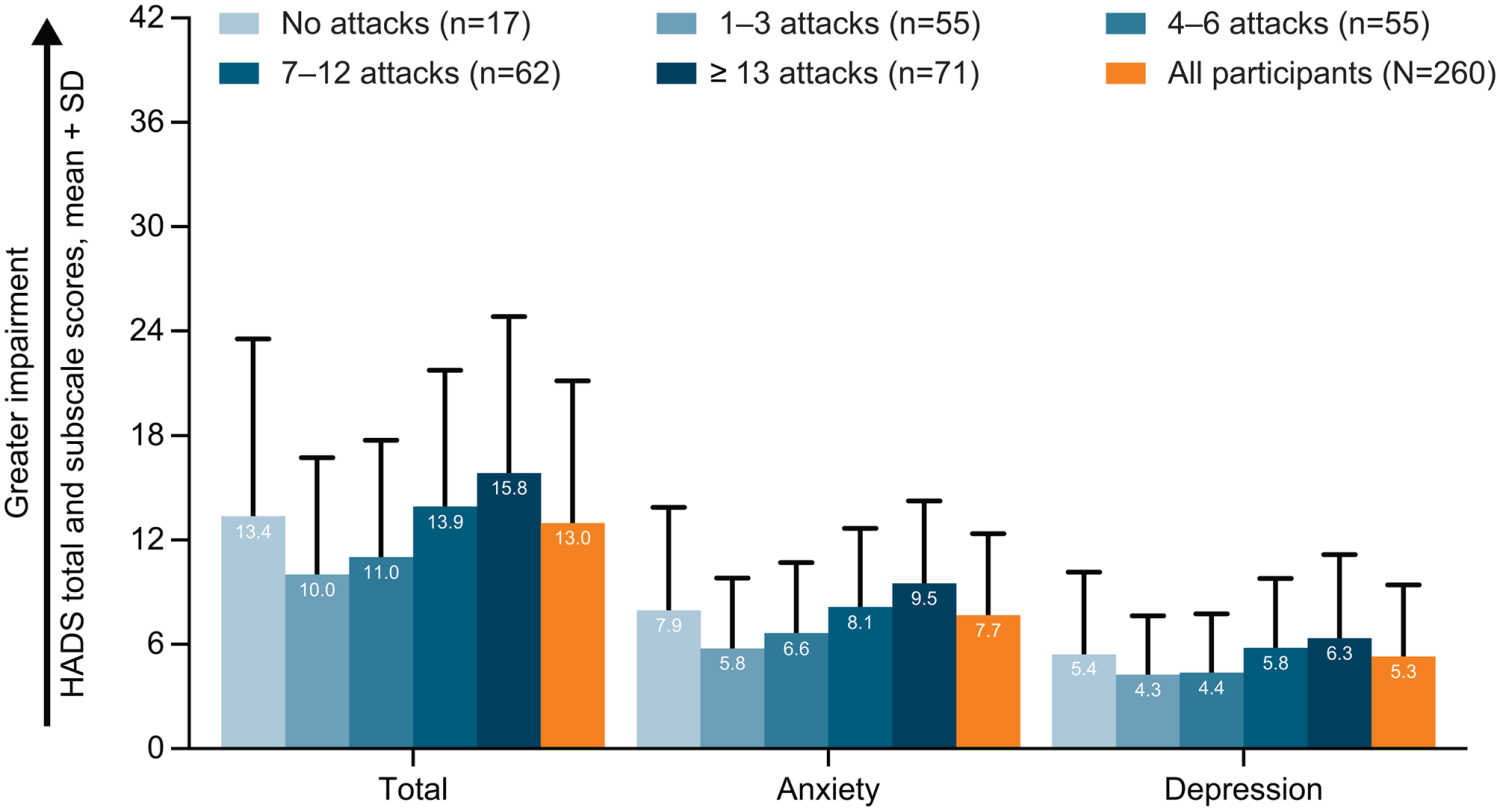
HADS total and subscale scores by the number of HAE attacks in the previous 6 months. *HADS* Hospital Anxiety and Depression Scale, *HAE* hereditary angioedema, *SD* standard deviation. Hospital Anxiety and Depression Scale (HADS) is a generic measure that assesses anxiety and depression which consists of 14 questions (7 for anxiety and 7 for depression) over a recall period “in the past week” [26]. The HADS anxiety and depression subscale scores have a scale of 0–21, where higher scores indicate a greater impairment [26]. HADS subscale scores of 0–7, 8–10, 11–14, and 15–21 indicate normal levels of anxiety or depression, mild anxiety or depression, moderate anxiety or depression, and severe anxiety or depression, respectively [26, 27]. HADS subscale scores can be summed to calculate the HADS total score, which reflects psychological distress and has a scale of 0–42 [28]

**Fig. 5:**
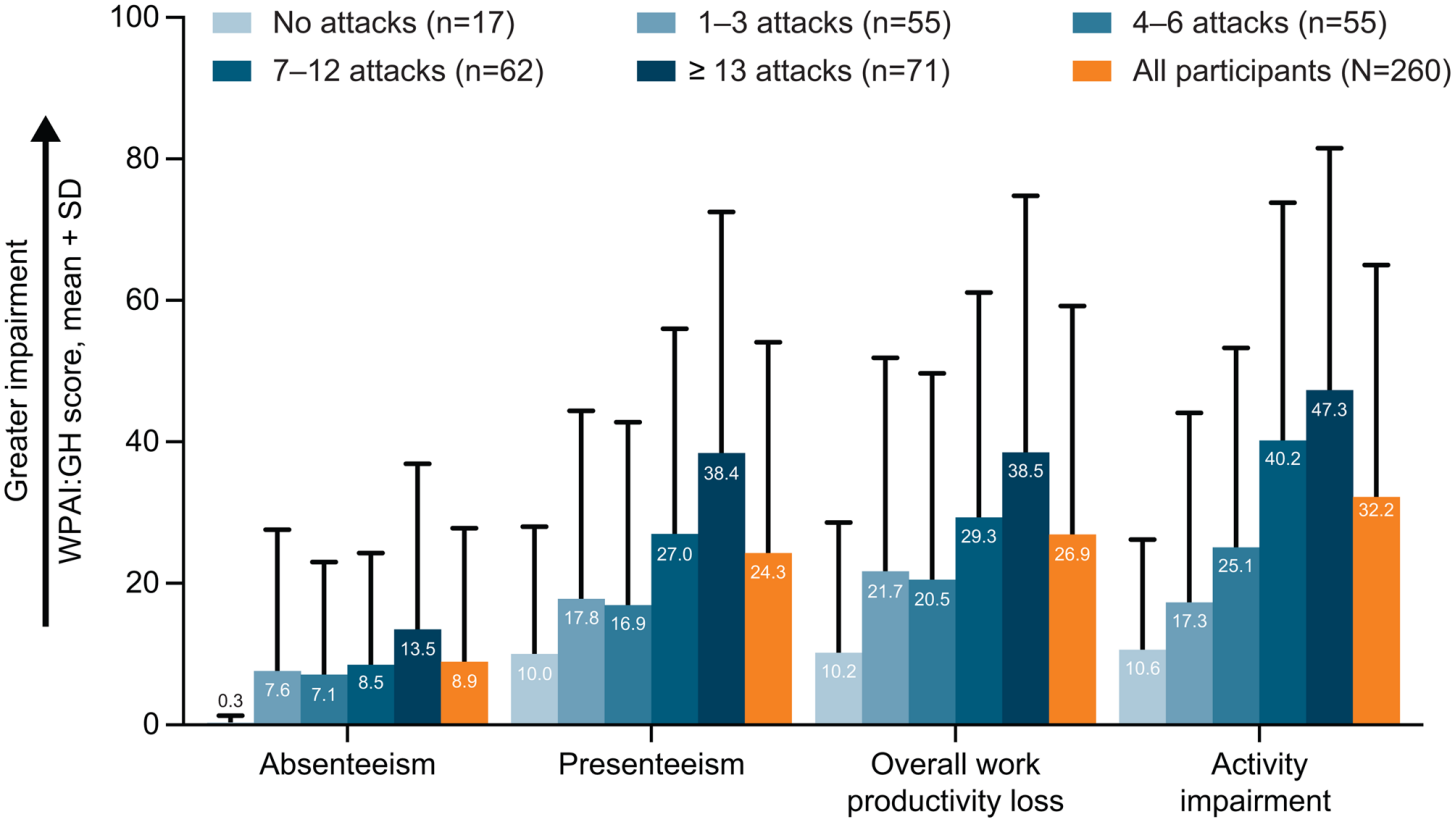
Percentage impairment measured by WPAI:GH by the number of HAE attacks in the previous 6 months. *HAE* hereditary angioedema, *SD* standard deviation, *WPAI:GH* Work Productivity and Activity Impairment: General Health. Work Productivity and Activity Impairment: General Health (WPAI:GH) is a generic measure that assesses health-related loss in work productivity which consists of 6 questions over a recall period “during the past 7 days” [29, 30]. WPAI:GH includes 4 domains (absenteeism, assessing work time missed; presenteeism, assessing reduced productivity while working; overall work productivity loss, assessing combined absenteeism and presenteeism; and activity impairment, assessing impairment in daily activities not related to work); each domain score is expressed as a percentage of productivity loss/impairment, where higher scores indicate a greater loss in productivity [30]

### Health-related quality of life

AE-QoL total score (mean ± SD) of 42.9 ± 23.2, exceeding the ≥ 39-point threshold indicating moderate to large HRQoL impairment, was reported in the overall cohort (Fig. 3). An AE-QoL total score of ≥ 39 was reported by 137 of 260 (52.7%) participants. AE-QoL domain scores (mean ± SD) ranged from 35.4 ± 27.3 for Functioning to 50.4 ± 28.3 for Fears/Shame. AE-QoL total and domain scores by country are reported in Additional file 2: Supplementary Table 4.

AE-QoL total and domain scores varied depending on the location of the most recent HAE attack. For example, participants with their most recent HAE attack in multiple locations reported a numerically higher AE-QoL total score (mean ± SD, 48.5 ± 23.8) compared with those who reported their most recent HAE attack affecting extremities or abdomen (Table 3). Of the AE-QoL domains, the Fears/Shame domain score was numerically higher than other domains in all attack location groups, with the exception of participants with a most recent attack location of the abdomen whose highest AE-QoL domain score was Fatigue/Mood.

**Table 3:** AE-QoL scores by the location of the most recent HAE attack

	Extremities (*n* = 54)	Head/Face (*n* = 12)	Throat/ Mouth (*n* = 7)	Trunk (*n* = 2)	Abdomen (*n* = 53)	Other (*n* = 18)	Multiple locations (*n* = 111)	None (*n* = 3)
**AE-QoL score**,** mean ± SD**								
Total	37.0 ± 21.1	41.7 ± 24.6	41.8 ± 23.1	80.2 ± 5.2	36.2 ± 20.0	45.8 ± 24.5	48.5 ± 23.8	27.9 ± 24.3
Functioning	28.5 ± 24.7	35.9 ± 27.3	31.3 ± 28.6	78.1 ± 4.4	26.3 ± 20.0	41.0 ± 34.8	42.5 ± 27.9	8.3 ± 14.4
Fatigue/Mood	35.3 ± 24.6	37.9 ± 29.5	45.7 ± 23.2	80.0 ± 7.1	41.1 ± 26.8	42.5 ± 24.6	47.0 ± 26.8	43.3 ± 42.5
Fears/Shame	48.1 ± 27.2	51.4 ± 30.0	49.4 ± 33.5	81.3 ± 26.5	39.5 ± 26.9	54.4 ± 28.6	55.8 ± 27.8	33.3 ± 31.5
Nutrition	25.0 ± 25.4	33.3 ± 33.9	30.4 ± 21.5	81.3 ± 8.8	34.0 ± 25.4	38.2 ± 30.8	41.9 ± 31.6	12.5 ± 12.5

All data presented in this table are self-reported by survey participants

*AE-QoL* Angioedema Quality of Life, *HAE* hereditary angioedema, *SD* standard deviation

AE-QoL total and domain scores increased with the number of HAE attacks experienced in the past 6 months, suggesting a greater HRQoL impairment with a higher frequency of attacks (Fig. 2). Regardless of the number of HAE attacks in the past 6 months, AE-QoL Fears/Shame domain score was numerically highest of the AE-QoL domain scores.

### General health status

The SF-12 v2 Physical Component Summary and Mental Component Summary scores (mean ± SD) were 45.9 ± 9.2 and 42.9 ± 11.8, respectively. SF-12 v2 scores by country are reported in Additional file 2: Supplementary Table 5. The SF-12 v2 domain scores (mean ± SD) in the overall cohort were 48.0 ± 10.2 for physical function, 44.8 ± 9.7 for role-physical, 42.3 ± 11.9 for bodily pain, 42.1 ± 10.8 for general health, 48.1 ± 10.2 for vitality, 41.5 ± 12.4 for role-emotional, 42.5 ± 11.9 for social functioning, and 44.0 ± 11.1 for mental health.

Across all attack frequency subgroups, mean Physical Component Summary scores remained higher than mean Mental Component Summary scores (Fig. 3).There was a trend of decrease in SF-12 v2 domain scores as attack frequency increased; however, participants who reported no attacks in the past 6 months (*n* = 17, smallest number of participants among HAE attack number subgroups) had numerically lower physical function, role-physical, general health, social functioning, and role-emotional domain scores compared with those who reported 1–3 attacks. Participants who reported ≥ 13 attacks had the lowest mean summary and domain scores (Fig. 3).

### Anxiety and depression

In the overall cohort, the HADS total score (mean ± SD) was 13.0 ± 8.2, and anxiety and depression subscale scores were 7.7 ± 4.7 and 5.3 ± 4.1, respectively (Fig. 4), with scores by country reported in Additional file 2: Supplementary Table 6. A total of 69 (26.5%) and 27 (10.4%) participants reported moderate to severe anxiety and depression as measured by HADS, respectively; proportions were higher in female versus male participants (moderate to severe anxiety: 33.9% [64/189] versus 7.0% [5/71], respectively; moderate to severe depression, 12.2% [23/189] versus 5.6% [4/71], respectively).

The mean HADS total score and subscale scores for anxiety and depression were numerically highest in participants reporting ≥ 13 attacks in the past 6 months (Fig. 4). Mean subscale scores for anxiety and depression were generally higher as the number of attacks experienced increased, although the mean scores were numerically higher in participants who reported no attacks versus those who reported 1–3 or 4–6 attacks in the past 6 months.

### Work productivity and activity impairment

Participants in the overall cohort reported 8.9% ± 18.9 (mean ± SD) absenteeism as measured by WPAI:GH. The percentage impairment (mean ± SD) measured by the WPAI:GH was 24.3% ± 29.8 for presenteeism, 26.9% ± 32.3 for overall work productivity loss, and 32.2% ± 32.8 for activity impairment (Fig. 5). The participant-reported work productivity loss as measured by WPAI:GH overall work productivity loss domain score (mean ± SD) notably varied between countries, from 9.2% ± 15.1 to 63.3% ± 31.9 (Additional file 2: Supplementary Table 7).

The mean WPAI:GH scores tended to numerically increase with increasing number of participant-reported HAE attacks in the past 6 months, suggesting greater productivity loss in participants with higher attack frequency. For each WPAI:GH domain, the scores (mean ± SD) were highest in participants who reported ≥ 13 attacks in the past 6 months (absenteeism, 13.5% ± 23.4, presenteeism, 38.4% ± 34.1; work productivity loss, 38.5% ± 36.3; activity impairment, 47.3% ± 34.2) and lowest in participants who reported experiencing no attacks in the past 6 months (absenteeism, 0.3% ± 1.0; presenteeism, 10.0% ± 18.0; work productivity loss, 10.2% ± 18.4; activity impairment, 10.6% ± 15.6).

### Healthcare resource utilization

In the overall cohort, 195 of 260 (75.0%) participants had visited allergists/immunologists in the past 6 months, with 2.0 ± 2.3 (mean ± SD) visits due to HAE-related reasons and of 1.9 ± 9.0 visits due to non-HAE-related reasons. Eighty-four (32.3%) participants reported visits to general practitioner, internist, or primary care physician, with 2.4 ± 10.3 (mean ± SD) and 2.6 ± 3.7 visits in the past 6 months due to HAE-related and non-HAE- related reasons, respectively. These types of providers were also the most visited by participants regardless of the frequency of attacks in the past 6 months. The number of visits to doctors regardless of type generally increased with the frequency of attacks. The most frequently visited medical professional for HAE-related reasons was a physician assistant or a nurse practitioner with 10.9 ± 23.2 (mean ± SD) visits in the past 6 months.

In the past 6 months, 232 (89.2%), 227 (87.3%) and 252 (96.9%) participants reported 0 or 1 visit to the emergency room, urgent care, and hospital, respectively. Six or more visits to emergency room, urgent care, and hospital were reported by four (1.5%), five (1.9%), and one (0.4%) participant, respectively. Participants with ≥ 1 visit to the emergency room due to HAE attacks (56/260; 21.5%) reported 2.3 ± 2.1 (mean ± SD) visits in the past 6 months, participants with ≥ 1 visit to urgent care due to HAE attacks (61/260; 23.5%) reported 2.5 ± 2.5 visits in the past 6 months, and participants with at ≥ 1 visit to hospital due to HAE attacks (27/260; 10.4%) reported 1.7 ± 1.5 visits in the past 6 months.

The number of visits to urgent care increased with the frequency of attacks; this trend applied to emergency room and hospital visits with two exceptions: participants who reported ≥ 13 attacks in the past 6 months reported a lower number of visits to the emergency room compared with those who reported 7–12 attacks, and participants who reported no attacks reported a higher number of hospital visits compared with those who reported 1–3 attacks. The average number of visits to these three healthcare settings in the past 6 months was consistently < 1 across all attack frequencies. The only exception was in visits to urgent care in participants who reported ≥ 13 attacks in the past 6 months, with 1.1 ± 2.5 (mean ± SD) visits reported.

## Discussion

This multinational study survey explored the burden of disease in participants with HAE from expanded geographical locations compared with the previous surveys. To our knowledge, the results of this study represent the first reported evaluation of burden of disease in patients with HAE from Argentina, Colombia, Croatia, Hungary, Ireland, Poland, Portugal, and Romania. The findings on the burden of disease are consistent with previous surveys conducted in the United States [16] and other high-income countries (Australia, Austria, Canada, France, Germany, Spain, Switzerland, and the United Kingdom) [14, 18], highlighting that many of the issues faced by the patients with HAE are not specific to a geographic area.

Most participants in this survey were female (despite equal HAE prevalence reported in males and females), aged approximately 40 years, and had family history of HAE; over half started experiencing HAE symptoms before the age of 12 and almost 80% started experiencing HAE symptoms before the age of 18. These demographic and clinical characteristics are typical to those reported in the literature [7, 17]. The fact that the majority of patients with a self-reported diagnosis of HAE-nC1INH were from Brazil is not unexpected, with a survey of HAE-treating physicians reporting that 40% of patients in Brazil had a diagnosis of HAE-nC1INH [31]. While the frequency and severity of attacks were highly variable, participants of this survey reported a mean of 11.5 attacks in the past 6 months. HAE attack frequency, areas affected by the most recent attack (mainly trunk and extremities), as well as the symptoms experienced by the participants from this survey were consistent with previous publications [4, 14–16]. In our study, 7.8% of participants reported the throat or larynx as the area affected in their most recent attack, similar to 6.6% reported in another study on the burden of HAE [14]; laryngeal attacks are of clinical importance as they can be life-threatening. The fatal nature of the disease should not be underestimated, as approximately a third (38.3%) of the survey respondents reported the loss of a family member due to suffocation related to an HAE attack.

LTP use was reported in approximately half of the survey participants, consistent with previous reports [4, 16]. Participants commonly reported the use of second-line LTP medications for HAE-C1INH (androgens and tranexamic acid); notably patients also reported the use of medications that were not indicated for use in HAE and excluded from international guidelines for the management of LTP of HAE attacks, for example loratadine and fexofenadine [1, 11]. Approximately one-third of patients with current LTP reported using androgens (56/153; 36.6%); while substantial androgen use in Brazil (reported by >70% of patients from Brazil who were using LTP), where access to first-line LTP may be more limited, is unsurprising, it was interesting to see widespread androgen use in Portugal (reported by >90% of patients from Portugal who were using LTP) and some use in other countries from Europe, including Hungary, Ireland, Croatia, and Poland. Although the efficacy of tranexamic acid as LTP in patients with HAE-C1INH is not firmly established, more than 10% of participants currently using LTP reported using tranexamic acid for LTP. The need for better access to modern, effective, and safe LTP medications may persist in some of the countries included in this survey, either due to lack of regulatory approval in specific countries or due to challenges in accessing approved medications through the local public health systems. For example, lanadelumab was approved by regulatory authorities in all the countries included in this survey before the study initiation, and berotralstat was approved in most of the countries (excluding Argentina, Brazil, and Colombia); however, the majority of participants who reported lanadelumab and berotralstat use were from Germany, Norway, and Sweden. These scenarios may be related to issues around reimbursement, with discussions often being multi-staged and occurring some time post approval [32]. Indeed, in a 2023 survey 73% and 51% of HAE-expert physicians reported treatment costs and reimbursement, respectively, as unmet treatment needs [33]. Reimbursement may also be limited to patients with particular disease characteristics. For example, following the approval of lanadelumab and berotralstat in Ireland, reimbursement was only available in patients with >2 attacks per week over an 8 week period despite oral LTP use that were not suitable for treatment with icatibant or C1INH on-demand medications [34], while in Poland reimbursement of lanadelumab was only available to approximately 50 patients between 2021 and 2023 [35].

This study used several validated PROs to measure disease control, HRQoL, general health status, anxiety, depression, and work performance. The survey participants reported moderate to large HRQoL impairment as measured by AE-QoL, consistent with previous burden of disease surveys in patients with HAE [4, 14, 16]. Furthermore, the mean AE-QoL total score in our survey (42.9) was similar to that observed at baseline of clinical studies in HAE (ranging between 36.8 and 48.8 across study arms from several studies) [36–39]. These findings suggest that HRQoL impairment in the participants from this survey was similar to that in patients enrolling in clinical studies for modern LTP medications, despite the potential differences in populations due to inclusion/exclusion criteria.

In our study, the mean AECT score was 7.4, and most participants reported a score of < 10, which indicates perception of poor disease control; these findings are aligned with the findings from another multinational survey [14]. Data on AECT at baseline of clinical studies, before treatment initiation, is scarce; however, in two studies of modern LTP medications with available AECT data post-treatment, >85% of patients report AECT scores ≥ 10 (perception of well-controlled disease) following treatment with modern LTP medications [20, 39, 40]. Furthermore, the clinical study data show the largest improvement following long-term modern LTP treatment in the AE-QoL Fears/Shame domain [40].

Although the participants from our study reported poorly controlled disease, high HAE attack rates, and the majority of participants expressed some degree of concern over the unpredictability of their HAE and the lack of control, a notable proportion of patients (approximately three quarters of those responding to the specific question) indicated being satisfied or very satisfied with their current treatment regimen. One of the possible explanations is that participants may be unaware of modern HAE medications and their potential benefits in disease control, leading to belief that their treatment regimen cannot be better than it is. LTPs with proven efficacy and safety may help to address patient concerns about the unpredictability of their disease and the fears and shame associated with HAE.

The mean HADS anxiety and depression subscale scores in the current study (7.7 for anxiety and 5.3 for depression, with a mean total score of 13.0) were indicative of normal to mild levels of anxiety and normal levels of depression, similar to the HADS scores at baseline in clinical HAE studies [40, 41]. However, 26.5% and 10.4% of participants from our survey reported moderate to severe anxiety and depression as measured by HADS, respectively, in agreement with the literature [14]; severe anxiety and depression as measured by HADS was reported by 9.2% and 2.7% of participants, respectively. Despite the mean HADS scores indicating normal to mild levels of anxiety and normal levels of depression, participants reported a notable impairment in fears and shame as measured by AE-QoL and mental burden as evaluated by the SF-12 v2 Mental Component Summary score. Mental burden in patients with HAE is supported by demographic data from our survey showing that 54 participants reported having anxiety and 34 reported taking prescription medication to treat anxiety, and 26 participants reported having depression and 19 reported taking prescription medication to treat depression. Notably, the HADS subscale scores indicated moderate to severe anxiety in 69 participants and moderate to severe depression in 27 participants.

Our study results also showed poorer general health versus the general population as measured by SF-12 v2 and notable productivity impairment as measured by WPAI:GH; these findings were aligned with the results from previous surveys in patients with HAE [4, 14, 16]. Furthermore, mean WPAI:GH scores in our study (absenteeism, 8.9%; presenteeism, 24.3%; overall work productivity loss, 26.9%; activity impairment, 32.2%) were similar to those reported as HAE clinical study baseline data (6.6% for absenteeism, 24.8% for presenteeism, 26.6% for overall work productivity loss, and 34.1% for activity impairment) [41].

Overall, our study results show that participants with a higher number of HAE attacks tended to report poorer HRQoL as measured by different PRO measures including AECT, AE-QoL, SF-12 v2, and WPAI:GH versus those with fewer attacks, consistent with results from previous burden of disease surveys [14, 16]. A clear trend between HADS scores and the number of HAE attacks was not observed, although proportions of participants with severe anxiety and depression were higher in the subgroup of participants who reported ≥ 13 versus fewer attacks in the past 6 months, in agreement with previous surveys [14, 16]. Given that the HADS is not specific to HAE, it may be that there were other factors not attributable to HAE that contributed to anxiety and depression among patients with relatively few HAE attacks. Nonetheless, our results suggest that participants with few HAE attacks continued to experience a degree of HRQoL impairment, which is potentially related to the unpredictability of their disease. Indeed, over 85% of participants reported disease unpredictability to be at least a little bothersome.

In agreement with our results, previous surveys have reported that most patients scheduled visits to allergists/immunologists for management of HAE [14]. These results are consistent with current clinical guideline recommendations to monitor disease activity, impact, and control in patients with HAE, especially those who are using LTP [1, 11]. Follow-up visits every 6 to 12 months are suggested for patients with well-controlled symptoms, while those with more frequent or disruptive HAE symptoms may require more frequent follow-up [13]. Similar to our study, a different survey of patients with HAE from Australia, Canada, and six countries in Europe reported visiting a physician assistant or a nurse practitioner for HAE-related issues [14]; the patients from the previous survey reported 22.5 visits to a physician assistant or nurse practitioner per year, similar to 10.9 visits in the past 6 months in the present study. Regarding the number of visits to the emergency room, two surveys in the United States reported averages of 4.7 and 5.2 visits per year [16, 17], which is higher than reported by patients in our study (0.5 in the past 6 months); however, it is unknown whether the causes of the visits in these two studies were HAE-related. Considering that the current study was conducted from July 2022 to February 2023, the lasting effects of the COVID-19 pandemic may have impacted the results associated with HCRU due to limited patient access to hospitals. Although the use of remote health services may have been impacted during the course of the COVID-19 pandemic, the survey did not have any questions to evaluate the use of remote health services. In a 2018 multinational survey of patients with HAE from Australia, Austria, Canada, France, Germany, Spain, Switzerland, and the United Kingdom, the mean number of visits to emergency rooms, hospitals, or urgent care clinics due to HAE attacks among the participants who reported a visit ranged from 5.2 to 5.7 in a year, compared with the current study (1.7 to 2.5 in the past 6 months) [14] [Takeda, unpublished data]. Meanwhile, the HRQoL reported in these two studies was similar. The HCRU in our study was low, taking into consideration that approximately 75% of participants reported that their last attack was moderate to severe.

The study had several limitations. Selection bias may have existed due to the route of recruitment via healthcare providers and patient associations. The inclusion criteria also meant that patients who were attack-free or asymptomatic were ineligible to participate, which may have introduced selection bias. Additionally, the survey relied on self-reported, retrospective data, which may have caused recall bias. Although the survey included multiple PROs to capture the patient-reported burden of disease, some limitations imposed by HAE may not have been captured by existing PROs, for example, types of activities limited by HAE. Further investigations are required to better understand the ways HAE may limit patient behavior beyond the burden of disease described by the existing PROs. The cross-sectional design of the study meant that it was not possible to assess changes in the burden of disease over time. Additionally, the cross-sectional design and absence of baseline and follow-up assessments precluded valid analysis of outcomes in subgroups defined by specific treatments participants were receiving, as this comparison requires longitudinal data to assess changes over time for their unbiased interpretation. It was also not possible to conduct correlation analyses between symptoms and attack locations, which could be of interest for future research.

The results of this study provide a comprehensive description of the burden of disease in participants with HAE across multiple countries. The survey was developed with all the necessary scientific rigor, including concept elicitation interviews with patients with HAE, cognitive interviewing following the development of the survey, multiple rounds of review by ethics committees both globally and locally for each site as required by local regulations, and alignment with local representatives of the study sponsor and within the local sites to ensure adequate recruitment. Furthermore, the PROs used in this study have previously been used in several clinical trials evaluating prophylactic treatments for patients with HAE [36–39, 41–43]. Further work may be required to understand the characteristics of participants from subgroups who are not optimally served. Additionally, future studies employing different methodologies could link the data between a patient survey and medical records to better elucidate HCRU and treatment patterns.

## Conclusions

The results of this large, multinational survey of 260 participants with HAE from 13 countries suggest that access to first-line LTP options was not universal, with only a third of participants on LTP reporting their use. The participants experienced high attack rates (approximately one attack every 2 weeks on average), impaired HRQoL, and notable anxiety and depression due to their HAE. AE-QoL, AECT, HADS, and WPAI:GH worsened, overall, as attack frequency increased; however, in our study, the number of attacks did not show a clear relationship with HCRU. Although the majority of participants had family members with HAE, the mean delay between HAE onset and HAE diagnosis exceeded 10 years in the participants from this survey. Overall, survey findings suggest that HAE continues to impose a substantial burden on patients, at least in part due to limited access to modern LTP medications.

## Supplementary Information

Below is the link to the electronic supplementary material.

Supplementary Material 1

Supplementary Material 2

## Data Availability

The datasets generated during and/or analyzed during the current study are available from the corresponding author on reasonable request.
