# Supplementary material for: Burden of disease in adult patients with hereditary angioedema: results from a multinational survey
Source: Orphanet J Rare Dis. 2026 Feb 19;21:70. doi: 10.1186/s13023-025-04134-z (PMC12922389; doi:10.1186/s13023-025-04134-z)
Supplement: Supplementary file 1 — Supplementary Material 1 [file 13023_2025_4134_MOESM1_ESM.pdf]

Burden of disease in people living with hereditary angioedema

This is a visual summary of an article titled "Burden of disease in adult patients with hereditary angioedema: results from a multinational survey," which was published in *Orphanet Journal of Rare Diseases* in 2025.

How to say:

- Angioedema: "an-jee-ow-uh-dee-muh"
- Berotralstat: "ber-oh-tral-stat"
- Danazol: "da-nuh-zole"
- Esterase: "eh-str-ayz"
- Hereditary : "hr-eh-duh-teh-ree"
- Inhibitor: "unh-hi-buh-tr"
- Lanadelumab: "lan-a-del-ue-mab"
- Oxandrolone: "ok-san-droh-lone"
- Prophylaxis: "prow-fuh-lak-suhs"
- Tranexamic: "tran-eks-am-ik"

What is hereditary angioedema (HAE for short)?

HAE is a rare genetic disease that is equally likely to affect men and women. Genetic diseases are caused by changes in a person's genes that can be passed from parents to children (hereditary) or develop spontaneously (on their own).

In most people with HAE, a genetic change affects a protein called C1 inhibitor (C1INH for short). C1INH is involved in regulating the flow of body fluids to and from cells. Low levels of C1INH, or C1INH that is not working properly, can cause swellings (known as angioedema) of the skin and deeper tissues, known as HAE attacks.

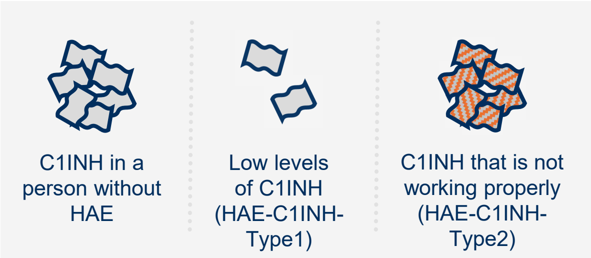

HAE attacks are unpredictable. Swelling is the most common in:

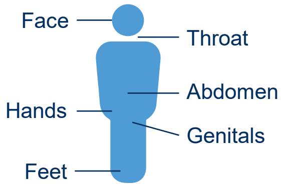

An attack affecting the airway can become life-threatening.

How often someone has an HAE attack, how serious they are, and how long they last will vary from person to person.

People with HAE may also experience other symptoms:

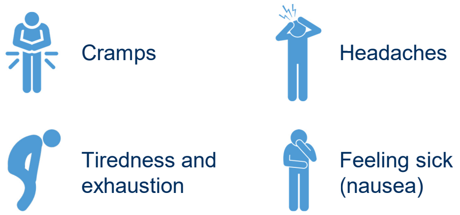

How is HAE treated?

People with HAE can either take medications when attacks occur, known as on-demand or rescue treatment, or take medications to prevent attacks from occurring, known as long-term preventative treatment (prophylaxis). The medicines for long-term preventative treatment recommended by international guidelines for doctors treating HAE include **human C1INH, lanadelumab**, and **berotralstat**. However, these medicines may not be available in all countries. When they are not available, people can receive other medicines for long-term preventative treatment, such as danazol, oxandrolone, or tranexamic acid.

Why was the study done?

The funder of the study, Takeda, wanted to better understand what is it like for individuals to live with HAE in different parts of the world.

What did the study look at?

- What were the survey participants' symptoms and HAE attacks like?
- Did the participants think their disease was under control?
- What was the quality of life like for participants with HAE?
- Did HAE affect participants' ability to work or do things that they would normally do?

Study plan and participants

Online survey between July 2022 and February 2023 in 13 countries

260 adults with HAE

71 men

189 women

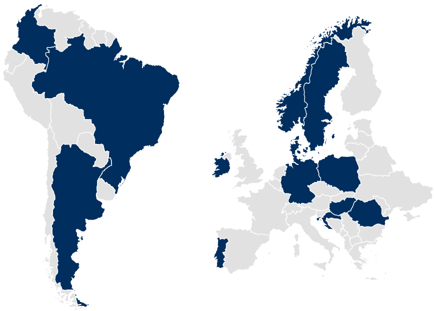

- Argentina
- Brazil
- Colombia
- Croatia
- Denmark
- Germany
- Hungary
- Ireland
- Norway
- Poland
- Portugal
- Romania
- Sweden

What was it like to live with HAE?

On average, the participants were:

- **12 years old** when they had their first HAE attack**;**
- **24 years old** when they were diagnosed with HAE.

Approximately **8 in every 10** participants had a family member with HAE.

On average, participants had experienced an average of 12 attacks in the last 6 months.

Approximately **4 in every 10** participants said that their most recent attack lasted over 24 hours.

The most recent HAE attack most frequently involved swelling in the stomach (approximately **5 in every 10** attacks) and limbs (feet in more than **2 in every 10** attacks and hands in approximately **2 in every 10** attacks).

Most participants considered that their most recent attack was **moderate** in severity.

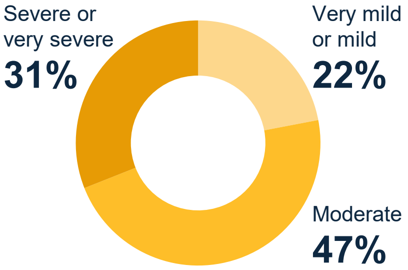

Approximately **6 in every 10** participants reported using long-term preventative treatment.

Of those using long-term preventative treatment:

Approximately **4 in every 10** reported using medications recommended by the guidelines.

Approximately **4 in every 10** reported using androgens (danazol and/or oxandrolone).

More than **7 in every 10** participants did not think their disease was under control.

Participants who reported currently taking long-term preventative treatment considered their disease being more under control than those who did not take long-term preventative treatment.

Approximately **5 in every 10** participants had quality of life worsened by a moderate to large amount.

Approximately **3 in every 10** participants had moderate to severe anxiety.

Approximately **1 in every 10** participants had moderate to severe depression.

Approximately **3 in every 10** participants were limited in their everyday activities.

Approximately **2 in every 10** participants were less productive at work.

Approximately **1 in every 10** participants missed work because of HAE.

Participants with more HAE attacks were more likely to have poorer health-related quality of life, anxiety and depression, and tiredness, and were less able to work or do things they would normally do.

What does this study tell us and why is it important?

The average delay from first HAE symptoms to a confirmed diagnosis of HAE was approximately 12 years.

Access to guideline-recommended long-term preventative treatments was not available for all people in the countries included in the survey.

Participants of this survey had frequent HAE attacks that often worsened their quality of life. For many, HAE caused anxiety and depression and/or limited some of their daily or work activities.

More information

More information about HAE can be found on the websites below:

HAE International: https://haei.org/

Genetic and Rare Diseases Information Center: https://rarediseases.info.nih.gov/diseases/5979/hereditary-angioedema

The study was funded by Takeda Development Center Americas, Inc. Medical writing support for this summary was provided by Claire Line, PhD, and Milda Tyler, PhD, of Excel Scientific Solutions, Inc.
The original authors of the full article reviewed and approved this summary.
