# Supplementary material for: Burden of disease in adult patients with hereditary angioedema: results from a multinational survey
Source: Orphanet J Rare Dis. 2026 Feb 19;21:70. doi: 10.1186/s13023-025-04134-z (PMC12922389; doi:10.1186/s13023-025-04134-z)
Supplement: Supplementary file 2 — Supplementary Material 2 [file 13023_2025_4134_MOESM2_ESM.docx]

Additional file 2: Supplementary Tables 1–7

**Burden of disease in adult patients with hereditary angioedema: results from a multinational survey**

Maureen Watt,^1^ Inmaculada Martinez‑Saguer,^2^ Angela Simon,^1^ Ryan Murphy,^3^ Marie De La Cruz,^3^ Ricardo Zwiener,^4^ Mauricio Sarrazola,^5^ Anete S. Grumach^6^

^1^Takeda Development Center Americas, Inc., Lexington, MA, USA; ^2^HZRM Hemophilia Center Rhine Main, Frankfurt/Main, Germany; ^3^ICON, Raleigh, NC, USA; ^4^Servicio de Alergia e Inmunología Clínica, Hospital Universitario Austral, Pilar, Buenos Aires, Argentina; ^5^Departamento de Medicina, Grupo GIPPAM, Universidad de Pamplona, Cúcuta, Colombia; ^6^Clinical Immunology, Faculdade de Medicina, Centro Universitario Faculdade de Medicina ABC (CEUFMABC), Santo Andre, Brazil

**Correspondence:**

**Name:** Maureen Watt

**Address:** Takeda Development Center Americas, Inc., 500 Kendall Street, Cambridge, MA 02142, USA

**Tel:** +1 617 784 9707

**E-mail:** maureen.watt@takeda.com

**Supplementary Table 1a** Participant demographic and clinical characteristics by country

| Characteristic | ARG n=45 | BRA n=53 | COL n=25 | CRO  n=5 | DEN  n=4 | GER n=30 | HUN n=26 | IRL  n=10 | NOR n=10 | POL n=16 | POR n=15 | ROU n=13 | SWE  n=8 | Total  N=260 |
| --- | --- | --- | --- | --- | --- | --- | --- | --- | --- | --- | --- | --- | --- | --- |
| Age, years |  |  |  |  |  |  |  |  |  |  |  |  |  |  |
| Mean ± SD | 41.8 ± 13.2 | 41.0 ± 13.6 | 38.4 ± 13.3 | 42.2 ±  7.6 | 52.5 ± 13.3 | 43.9 ± 14.5 | 44.5 ± 14.9 | 45.9 ± 12.6 | 47.8 ± 13.2 | 44.3 ± 12.9 | 49.8 ± 13.4 | 42.5 ± 10.2 | 48.4 ± 14.3 | 43.3 ±  13.5 |
| Range | 18.0–  71.0 | 18.0–  66.0 | 18.0–  73.0 | 32.0–  51.0 | 37.0–  69.0 | 19.0–  67.0 | 19.0–  75.0 | 30.0–  65.0 | 28.0–  67.0 | 22.0–  66.0 | 28.0–  81.0 | 19.0–  60.0 | 32.0–  72.0 | 18.00–81.0 |
| Sex, n (%) |  |  |  |  |  |  |  |  |  |  |  |  |  |  |
| Female | 37 (82.2) | 43 (81.1) | 16 (64.0) | 4 (80.0) | 2 (50.0) | 17 (56.7) | 18 (69.2) | 6 (60.0) | 8 (80.0) | 10 (62.5) | 12 (80.0) | 8 (61.5) | 8 (100.0) | 189 (72.7) |
| Male | 8 (17.8) | 10 (18.9) | 9 (36.0) | 1 (20.0) | 2 (50.0) | 13 (43.3) | 8 (30.8) | 4 (40.0) | 2 (20.0) | 6 (37.5) | 3 (20.0) | 5 (38.5) | 0 (0.0) | 71 (27.3) |
| HAE type, n (%) |  |  |  |  |  |  |  |  |  |  |  |  |  |  |
| HAE-C1INH^a^ | 44 (97.8) | 37 (69.8) | 22 (88.0) | 3 (60.0) | 4 (100.0) | 29 (96.7) | 25 (96.2) | 10 (100.0) | 9 (90.0) | 15 (93.8) | 14 (93.3) | 13 (100.0) | 8 (100.0) | 233 (89.6) |
| HAE-nC1INH | 0 (0.0) | 12 (22.6) | 1 (4.0) | 1 (20.0) | 0 (0.0) | 0 (0.0) | 0 (0.0) | 0 (0.0) | 1 (10.0) | 0 (0.0) | 0 (0.0) | 0 (0.0) | 0 (0.0) | 15 (5.8) |
| Unknown^b^ | 1 (2.2) | 4 (7.5) | 2 (8.0) | 1 (20.0) | 0 (0.0) | 1 (3.3) | 1 (3.8) | 0 (0.0) | 0 (0.0) | 1 (6.3) | 1 (6.7) | 0 (0.0) | 0 (0.0) | 12 (4.6) |
| Age at HAE onset, years, mean ± SD | 13.5 ±  8.9 | 12.9 ± 10.8 | 11.5 ±  5.6 | 14.2 ±  6.2 | 9.3 ±  4.0 | 11.5 ± 10.7 | 10.8 ±  8.3 | 8.9 ±  7.6 | 8.6 ±  5.4 | 12.3 ±  9.2 | 12.5 ± 10.1 | 14.2 ±  8.6 | 9.8 ±  4.7 | 12.0 ±  8.9 |
| Age at HAE diagnosis, years, mean ± SD | 26.8 ± 13.7 | 27.8 ± 13.2 | 28.2 ± 14.5 | 33.6 ±  11.5 | 23.8 ±  17.6 | 19.4 ± 14.1 | 18.2 ±  9.2 | 18.5 ± 15.4 | 25.4 ± 15.3 | 21.9 ± 12.1 | 21.5 ± 14.4 | 27.5 ±  9.7 | 13.3 ±  8.3 | 24.2 ±  13.6 |
| Family history of HAE, n (%) |  |  |  |  |  |  |  |  |  |  |  |  |  |  |
| Yes | 35 (77.8) | 40 (75.5) | 19 (76.0) | 2 (40.0) | 3 (75.0) | 24 (80.0) | 22 (84.6) | 8 (80.0) | 8 (80.0) | 12 (75.0) | 15 (100.0) | 8 (61.5) | 8 (100.0) | 204 (78.5) |
| No | 8 (17.8) | 9 (17.0) | 4 (16.0) | 2 (40.0) | 1 (25.0) | 5 (16.7) | 4 (15.4) | 2 (20.0) | 2 (20.0) | 3 (18.8) | 0 (0.0) | 4 (30.8) | 0 (0.0) | 44 (16.9) |
| Not sure | 2 (4.4) | 4 (7.5) | 2 (8.0) | 1 (20.0) | 0 (0.0) | 1 (3.3) | 0 (0.0) | 0 (0.0) | 0 (0.0) | 1 (6.3) | 0 (0.0) | 1 (7.7) | 0 (0.0) | 12 (4.6) |
| HAE attacks in the past 6 months, mean ± SD | 15.2 ± 15.2 | 7.2 ±  6.6 | 10.8 ± 12.1 | 51.8 ± 45.6 | 5.5 ±  5.7 | 7.3 ±  5.3 | 16.1 ± 13.4 | 3.0 ±  2.9 | 11.1 ±  8.8 | 12.7 ± 19.4 | 5.1 ±  4.9 | 20.3 ± 13.1 | 5.6 ±  4.8 | 11.5 ±  14.2 |

All data presented in this table are self-reported by survey participants

*ARG* Argentina, *BRA* Brazil, *COL* Colombia, *CRO* Croatia, *DEN* Denmark, *GER* Germany, *HAE* hereditary angioedema, *HAE-C1INH* hereditary angioedema due to C1 inhibitor deficiency; *HAE-nC1INH* hereditary angioedema due to normal C1 inhibitor, *HUN* Hungary, *IRL* Ireland, *LTP* long-term prophylaxis, *NOR* Norway, *POL* Poland, *POR* Portugal, *ROU* Romania, *SD* standard deviation, *SWE* Sweden

^a^Includes participants who answered “HAE Type I”, “HAE Type II”, or “Unsure of exact HAE type, but it is either HAE Type I or II” to the survey question “Which type of HAE do you have?”

^b^Includes participants who answered “I don’t know what type of HAE” to the survey question “Which type of HAE do you have?”

**Supplementary Table 1b** Current LTP treatment by country

|  | ARG n=45 | BRA n=53 | COL n=25 | CRO  n=5 | DEN  n=4 | GER n=30 | HUN n=26 | IRL  n=10 | NOR n=10 | POL n=16 | POR n=15 | ROU n=13 | SWE  n=8 | Total  N=260 |
| --- | --- | --- | --- | --- | --- | --- | --- | --- | --- | --- | --- | --- | --- | --- |
| Current medication used for LTP,^a^ n/N (%) |  |  |  |  |  |  |  |  |  |  |  |  |  |  |
| Any | 22/45 (48.9) | 42/53 (79.2) | 14/25 (56.0) | 3/5  (60.0) | 3/4  (75.0) | 12/30 (40.0) | 11/26 (42.3) | 9/10 (90.0) | 9/10 (90.0) | 5/16 (31.3) | 12/15 (80.0) | 4/13 (30.8) | 7/8  (87.5) | 153/260 (58.8) |
| Androgens |  |  |  |  |  |  |  |  |  |  |  |  |  |  |
| Danazol | 0 | 8/42 (19.0) | 5/14 (35.7) | 1/3  (33.3) | 0 | 0 | 4/11 (36.4) | 4/9  (44.4) | 0 | 1/5  (20.0) | 11/12 (91.7) | 0 | 0 | 34/153 (22.2) |
| Oxandrolone | 0 | 23/42 (54.8) | 0 | 0 | 0 | 0 | 0 | 0 | 0 | 0 | 0 | 0 | 0 | 23/153 (15.0) |
| C1INH |  |  |  |  |  |  |  |  |  |  |  |  |  |  |
| Human C1INH (Berinert) | 10/22 (45.5) | 1/42  (2.4) | 1/14  (7.1) | 0 | 0 | 5/12 (41.7) | 4/11 (36.4) | 0 | 2/9  (22.2) | 0 | 0 | 0 | 1/7  (14.3) | 24/153 (15.7) |
| Human C1INH (Cinryze) | 0 | 1/42  (2.4) | 0 | 0 | 0 | 0 | 0 | 0 | 0 | 0 | 0 | 0 | 0 | 1/153 (0.7) |
| Lanadelumab^b^ | 0^b^ | 0 | 4/14 (28.6) | 0 | 3/3 (100.0) | 8/12 (66.7) | 0^b^ | 2/9  (22.2) | 0 | 1/5  (20.0) | 1/12  (8.3) | 0 | 5/7  (71.4) | 24/153 (15.7) |
| Tranexamic acid | 0 | 12/42 (28.6) | 5/14 (35.7) | 1/3  (33.3) | 0 | 0 | 0 | 3/9 (33.3) | 0 | 0 | 0 | 0 | 0 | 21/153 (13.7) |
| Berotralstat | 0 | 0 | 0 | 0 | 0 | 0 | 0 | 0 | 8/9  (88.9) | 0 | 0 | 0 | 2/7  (28.6) | 10/153 (6.5) |
| Other^b^ | 14/22 (63.6)^b^ | 3/42  (7.1) | 1/14  (7.1) | 1/3  (33.3) | 0 | 0 | 4/11 (36.4)^b^ | 0 | 0 | 3/5  (60.0) | 0 | 4/4 (100.0) | 0 | 30/153 (19.6) |
| Missing^c^ | 23 | 11 | 11 | 2 | 1 | 18 | 15 | 1 | 1 | 11 | 3 | 9 | 1 | 107 |

All data presented in this table are self-reported by survey participants

*ARG* Argentina, *BRA* Brazil, *C1INH* C1 inhibitor, *COL* Colombia, *CRO* Croatia, *DEN* Denmark, *GER* Germany, *HUN* Hungary, *IRL* Ireland, *LTP* long-term prophylaxis, *NOR* Norway, *POL* Poland, *POR* Portugal, *ROU* Romania, *SD* standard deviation, *SWE* Sweden

^a^Participants could select more than 1 response (categories nonexclusive) and may be counted in more than 1 category

^b^Five patients from Argentina and three patients from Hungary who reported using “other” LTP were assumed to be receiving lanadelumab (e.g., verbatim text “Taxairo”, “Takzhyro”, or “Lanadelumab” in the free-text entry for “other LTP”)

^c^No response was given to the survey question on current LTP medication

**Supplementary Table 2a** Timing and duration of the most recent HAE attack

| Timing of the most recent HAE attack, n (%) | All participants  (N=260) |
| --- | --- |
| Within the last 7 days | 90 (34.6) |
| 1 to 2 weeks ago | 41 (15.8) |
| 2 to 3 weeks ago | 27 (10.4) |
| 3 to 4 weeks ago | 20 (7.7) |
| 1 to 2 months ago | 33 (12.7) |
| 2 to 3 months ago | 9 (3.5) |
| 3 to 4 months ago | 11 (4.2) |
| 4 to 5 months ago | 4 (1.5) |
| 5 to 6 months ago | 10 (3.8) |
| 6 to 7 months ago | 2 (0.8) |
| 7 to 8 months ago | 3 (1.2) |
| 8 to 9 months ago | 1 (0.4) |
| 9 to 10 months ago | 1 (0.4) |
| 10 to 11 months ago | 2 (0.8) |
| 11 to 12 months ago | 3 (1.2) |
| >12 months ago | 2 (0.8) |
| Don’t know/don’t remember | 1 (0.4) |
| **Duration of the most recent angioedema attack, n/N (%)** | **n=257** |
| Up to 6 hours | 48/257 (18.7) |
| Between 6 and 12 hours | 47/257 (18.3) |
| Between 12 and 24 hours or 1 day | 54/257 (21.0) |
| Between 1 and 2 days | 45/257 (17.5) |
| Between 2 and 3 days | 41/257 (16.0) |
| >3 days | 20/257 (7.8) |
| Don’t know/Don’t remember | 2/257 (0.8) |

All data presented in this table are self-reported by survey participants

**Supplementary Table 2b** Severity and location of the most recent HAE attack

| Severity of the most recent attack, n/N (%) | n=257 |
| --- | --- |
| None | 2/257 (0.8) |
| Very mild | 14/257 (5.4) |
| Mild | 42/257 (16.3) |
| Moderate | 120/257 (46.7) |
| Severe | 69/257 (26.8) |
| Very severe | 10/257 (3.9) |
| **Areas of the body affected by the most recent attack,^a^ n/N (%)** | **n=257** |
| Abdomen (stomach) | 127/257 (49.4) |
| Feet | 63/257 (24.5) |
| Hands | 59/257 (23.0) |
| Legs (including the joints) | 46/257 (17.9) |
| Arms (including the joints) | 45/257 (17.5) |
| Bowels or rectum | 42/257 (16.3) |
| Genitals | 38/257 (14.8) |
| Buttocks | 25/257 (9.7) |
| Bladder | 22/257 (8.6) |
| Throat or larynx (voice box) | 20/257 (7.8) |
| Back | 17/257 (6.6) |
| Lips | 17/257 (6.6) |
| Eyes | 14/257 (5.4) |
| Cheeks | 12/257 (4.7) |
| Chest | 12/257 (4.7) |
| Neck | 12/257 (4.7) |
| Side of the body (between chest and hip) | 12/257 (4.7) |
| Tongue | 9/257 (3.5) |
| Ears | 5/257 (1.9) |
| Uvula | 5/257 (1.9) |
| Other | 3/257 (1.2) |

All data presented in this table are self-reported by survey participants

^a^Participants could select more than 1 response (categories nonexclusive) and may be counted in more than 1 category

**Supplementary Table 2c** Symptoms reported during most recent HAE attack

| Symptoms reported during the most recent attack,^a^ n/N (%) | n=257 |
| --- | --- |
| Abdominal pain | 127/257 (49.4) |
| Swelling in the arms, legs, hands, or feet | 123/257 (47.9) |
| Abdominal swelling | 121/257 (47.1) |
| Tiredness | 100/257 (38.9) |
| Nausea | 73/257 (28.4) |
| Changes in mood | 64/257 (24.9) |
| Diarrhea | 58/257 (22.6) |
| Dizziness/Lightheadedness | 45/257 (17.5) |
| Difficulty using feet | 42/257 (16.3) |
| Swelling in the genitals | 40/257 (15.6) |
| Swelling in the bowels or rectum | 39/257 (15.2) |
| Headache | 39/257 (15.2) |
| Difficulty using hands | 31/257 (12.1) |
| Vomiting | 29/257 (11.3) |
| Constipation | 27/257 (10.5) |
| Swelling in the buttocks | 26/257 (10.1) |
| Pain (not including abdominal pain) | 26/257 (10.1) |
| Swelling in the throat and mouth | 24/257 (9.3) |
| Swelling in the trunk | 23/257 (8.9) |
| Difficulty swallowing | 20/257 (7.8) |
| Swelling in the head or face | 18/257 (7.0) |
| Voice change | 18/257 (7.0) |
| Difficulty with urinating | 16/257 (6.2) |
| Difficulty with breathing | 14/257 (5.4) |
| Other | 8/257 (3.1) |

All data presented in this table are self-reported by survey participants

*HAE* hereditary angioedema

^a^Participants could select more than 1 response (categories nonexclusive) and may be counted in more than 1 category

**Supplementary Table 3.** AECT scores by country

|  | ARG n=45 | BRA n=53 | COL n=25 | CRO n=5 | DEN n=4 | GER n=30 | HUN n=26 | IRL n=10 | NOR n=10 | POL n=16 | POR n=15 | ROU n=13 | SWE n=8 | Total N=260 |
| --- | --- | --- | --- | --- | --- | --- | --- | --- | --- | --- | --- | --- | --- | --- |
| AECT score,  mean ± SD | 6.0 ± 3.0 | 7.8 ± 2.5 | 7.2 ± 2.6 | 5.4 ± 3.4 | 9.0 ± 1.4 | 8.0 ± 2.3 | 8.1 ± 3.0 | 11.4 ± 2.6 | 7.1 ± 3.0 | 7.1 ± 3.9 | 8.6 ± 2.6 | 3.6 ± 2.3 | 8.6 ± 2.8 | 7.4 ± 3.1 |

*AECT* Angioedema Control Test, *ARG* Argentina, *BRA* Brazil, *COL* Colombia, *CRO* Croatia, *DEN* Denmark, *GER* Germany, *HUN* Hungary, *IRL* Ireland, *NOR* Norway, *POL* Poland, *POR* Portugal, *ROU* Romania, *SD* standard deviation, *SWE* Sweden

**Supplementary Table 4.** AE-QoL scores by country

|  | ARG n=45 | BRA n=53 | COL n=25 | CRO n=5 | DEN n=4 | GER n=30 | HUN n=26 | IRL n=10 | NOR n=10 | POL n=16 | POR n=15 | ROU n=13 | SWE n=8 | Total N=260 |
| --- | --- | --- | --- | --- | --- | --- | --- | --- | --- | --- | --- | --- | --- | --- |
| AE-QoL score, mean ± SD |  |  |  |  |  |  |  |  |  |  |  |  |  |  |
| Total | 47.1 ± 23.0 | 49.0 ± 22.0 | 50.3 ± 20.6 | 67.4 ± 33.8 | 21.3 ±  9.0 | 32.4 ± 13.0 | 32.0 ± 17.3 | 31.3 ± 17.9 | 41.2 ± 22.9 | 40.7 ± 25.7 | 35.9 ± 31.8 | 61.4 ± 24.9 | 31.1 ± 13.7 | 42.9 ± 23.2 |
| Functioning | 40.8 ± 27.5 | 34.3 ± 26.1 | 44.3 ± 25.2 | 62.5 ± 38.5 | 10.9 ±  7.9 | 28.1 ± 25.4 | 26.7 ± 22.2 | 21.9 ± 22.5 | 40.0 ± 24.0 | 37.5 ± 28.2 | 29.6 ± 34.5 | 57.7 ± 24.0 | 18.0 ± 16.2 | 35.4 ± 27.3 |
| Fatigue/Mood | 42.8 ± 28.0 | 46.0 ± 26.3 | 53.6 ± 24.7 | 60.0 ± 37.9 | 28.8 ± 19.3 | 38.0 ± 21.8 | 33.5 ± 21.7 | 33.5 ± 27.2 | 49.5 ± 27.4 | 41.9 ± 29.9 | 29.3 ± 28.9 | 56.2 ± 29.6 | 41.9 ± 16.7 | 42.8 ± 26.6 |
| Fears/Shame | 56.2 ± 26.8 | 64.2 ± 27.0 | 53.5 ± 21.3 | 78.3 ± 32.6 | 26.0 ± 13.3 | 35.3 ± 18.1 | 32.5 ± 25.2 | 41.3 ± 23.3 | 35.4 ± 20.0 | 46.4 ± 28.5 | 46.9 ± 37.8 | 71.2 ± 28.1 | 35.4 ± 19.8 | 50.4 ± 28.3 |
| Nutrition | 43.3 ± 29.0 | 39.6 ± 31.3 | 44.5 ± 28.9 | 62.5 ± 30.6 | 9.4 ±  12.0 | 17.9 ± 20.7 | 37.0 ± 26.8 | 15.0 ± 17.5 | 40.0 ± 35.3 | 27.3 ± 22.0 | 31.7 ± 31.7 | 52.9 ± 32.7 | 17.2 ± 14.9 | 35.8 ± 29.5 |

All data presented in this table are self-reported by survey participants

*AE-QoL* Angioedema Quality of Life, *ARG* Argentina, *BRA* Brazil, *COL* Colombia, *CRO* Croatia, *DEN* Denmark, *GER* Germany, *HUN* Hungary, *IRL* Ireland, *NOR* Norway, *POL* Poland, *POR* Portugal, *ROU* Romania, *SD* standard deviation, *SWE* Sweden

**Supplementary Table 5** SF-12 v2 summary scores by country

|  | ARG n=45 | BRA n=53 | COL n=25 | CRO n=5 | DEN n=4 | GER n=30 | HUN n=26 | IRL n=10 | NOR n=10 | POL n=16 | POR n=15 | ROU n=13 | SWE n=8 | Total N=260 |
| --- | --- | --- | --- | --- | --- | --- | --- | --- | --- | --- | --- | --- | --- | --- |
| Physical Component Summary score, mean ± SD | 45.5 ±  9.6 | 46.4 ±  9.0 | 45.7 ±  9.4 | 37.4 ±  6.5 | 52.8 ±  4.9 | 47.1 ±  8.5 | 49.9 ±  6.8 | 47.0 ±  8.3 | 43.6 ± 13.0 | 46.6 ±  7.1 | 46.9 ±  9.8 | 34.8 ±  7.0 | 45.1 ±  7.2 | 45.9 ±  9.2 |
| Mental Component Summary score, mean ± SD | 41.9 ±  9.5 | 40.6 ± 12.5 | 43.1 ±  9.2 | 34.6 ± 21.4 | 47.8 ±  8.7 | 43.8 ± 10.4 | 43.0 ± 15.5 | 46.2 ±  9.3 | 40.2 ± 14.4 | 46.3 ± 12.0 | 47.5 ± 11.5 | 42.0 ± 11.4 | 47.7 ± 10.8 | 42.9 ± 11.8 |

*ARG* Argentina, *BRA* Brazil, *COL* Colombia, *CRO* Croatia, *DEN* Denmark, *GER* Germany, *HUN* Hungary, *IRL* Ireland, *NOR* Norway, *POL* Poland, *POR* Portugal, *ROU* Romania, SF-12 v2, 12-Item Short Form Survey, *SD* standard deviation, *SWE* Sweden

**Supplementary Table 6** HADS scores by country

|  | ARG n=45 | BRA n=53 | COL n=25 | CRO n=5 | DEN n=4 | GER n=30 | HUN n=26 | IRL n=10 | NOR n=10 | POL n=16 | POR n=15 | ROU n=13 | SWE n=8 | Total N=260 |
| --- | --- | --- | --- | --- | --- | --- | --- | --- | --- | --- | --- | --- | --- | --- |
| HADS total score, mean ± SD | 13.2 ±  7.2 | 15.8 ±  7.5 | 12.1 ±  7.9 | 21.2 ± 14.6 | 6.5 ±  5.1 | 11.2 ±  7.8 | 11.4 ±  8.9 | 11.6 ± 10.1 | 11.8 ±  7.8 | 12.9 ±  9.7 | 9.2 ±  6.8 | 16.0 ±  6.3 | 11.0 ±  7.5 | 13.0 ±  8.2 |
| Anxiety subscale score, mean ± SD | 8.0 ± 4.2 | 9.2 ± 4.4 | 6.8 ± 4.1 | 12.0 ± 8.4 | 2.8 ± 1.7 | 6.6 ± 4.0 | 6.7 ± 4.4 | 6.8 ± 6.5 | 6.1 ± 4.4 | 7.5 ± 5.9 | 5.9 ± 4.2 | 10.3 ± 3.8 | 7.1 ± 4.7 | 7.7 ± 4.7 |
| Depression subscale score, mean ± SD | 5.1 ± 3.7 | 6.6 ± 3.8 | 5.3 ± 4.2 | 9.2 ± 6.3 | 3.8 ± 4.3 | 4.6 ± 4.1 | 4.7 ± 5.1 | 4.8 ± 4.0 | 5.7 ± 4.8 | 5.4 ± 4.3 | 3.3 ± 2.9 | 5.7 ± 3.6 | 3.9 ± 3.6 | 5.3 ± 4.1 |

All data presented in this table are self-reported by survey participants

*ARG* Argentina, *BRA* Brazil, *COL* Colombia, *CRO* Croatia, *DEN* Denmark, *GER* Germany, *HADS* Hospital Anxiety and Depression Scale, *HUN* Hungary, *IRL* Ireland, *NOR* Norway, *POL* Poland, *POR* Portugal, *ROU* Romania, *SD* standard deviation, *SWE* Sweden

**Supplementary Table 7** WPAI:GH scores by country

|  | ARG n=45 | BRA n=53 | COL n=25 | CRO n=5 | DEN n=4 | GER n=30 | HUN n=26 | IRL n=10 | NOR n=10 | POL n=16 | POR n=15 | ROU n=13 | SWE n=8 | Total  N=260 |
| --- | --- | --- | --- | --- | --- | --- | --- | --- | --- | --- | --- | --- | --- | --- |
| WPAI:GH percent impairment,  mean ± SD |  |  |  |  |  |  |  |  |  |  |  |  |  |  |
| Absenteeism | 23.6 ± 28.0 | 8.8 ±  20.3 | 8.7 ±  13.4 | 20.0 ± 40.0 | 2.6 ±  4.6 | 5.7 ±  14.3 | 1.7 ±  7.5 | 4.6 ±  10.0 | 0.0 ±  0.0 | 0.0 ±  0.0 | 1.6 ±  3.3 | 17.9 ± 17.7 | 4.5 ±  8.7 | 8.9 ± 18.9 |
| Presenteeism | 37.3 ± 31.8 | 30.3 ± 33.5 | 32.1 ± 36.4 | 40.0 ± 45.5 | 10.0 ± 17.3 | 9.2 ±  16.1 | 11.1 ± 20.0 | 16.7 ± 25.8 | 16.0 ± 23.0 | 10.0 ± 15.5 | 8.0 ±  11.4 | 55.0 ± 33.8 | 42.0 ± 11.0 | 24.3 ± 29.8 |
| Overall work productivity loss | 45.7 ± 34.9 | 31.4 ± 35.7 | 34.3 ± 36.5 | 40.0 ± 45.5 | 11.8 ± 20.5 | 13.6 ± 22.0 | 10.3 ± 20.5 | 19.0 ± 29.6 | 13.3 ± 21.6 | 9.2 ±  15.1 | 9.3 ±  13.0 | 63.3 ± 31.9 | 44.3 ± 13.6 | 26.9 ± 32.3 |
| Activity impairment | 42.7 ± 33.9 | 39.1 ± 35.3 | 32.0 ± 31.0 | 54.0 ± 45.6 | 12.5 ± 15.0 | 16.3 ± 22.5 | 16.5 ± 23.7 | 12.0 ± 16.2 | 39.0 ± 34.8 | 23.1 ± 33.2 | 20.0 ± 29.0 | 65.4 ± 26.3 | 37.5 ± 28.2 | 32.2 ± 32.8 |

All data presented in this table are self-reported by survey participants

*ARG* Argentina, *BRA* Brazil, *COL* Colombia, *CRO* Croatia, *DEN* Denmark, *GER* Germany, *HUN* Hungary, *IRL* Ireland, *NOR* Norway, *POL* Poland, *POR* Portugal, *ROU* Romania, *SD* standard deviation, *SWE* Sweden, *WPAI:GH* Work Productivity and Activity Impairment: General Health
